# Genital rash as an initial presentation of monkeypox^☆^

**DOI:** 10.1016/j.abd.2022.09.001

**Published:** 2022-11-11

**Authors:** Milan Bjekic, Milica Markovic, Lidija Dejanovic

**Affiliations:** a City Institute for Skin and Venereal Diseases, Belgrade, Serbia; b Faculty of Dentistry, Pancevo, Belgrade, Serbia

Dear Editor,

Monkeypox is an endemic disease of Central and Western Africa caused by the Monkeypox virus, a member of the Orthopoxvirus genus. The disease was first diagnosed in humans in 1970 in a baby in Zaire^1^ and since then, cases in persons outside Africa have been often linked to international travel or contact with imported animals.^2^, ^3^ Recently, an outbreak of monkeypox has occurred worldwide.^4^ In the European region, on 22 June 2022, the European Centres for Disease Control identified a total number of 2746 cases from 29 countries, including the Republic of Serbia.^5^ Herein, we report a case of monkeypox with genital rash mimicking sexually transmitted infection.

A 35-year-old man was referred to the Department of Sexually Transmitted Infections for the evaluation of a painful genital rash that had appeared five days earlier and was followed by fever. His personal history showed that fever and skin lesions appeared 5 days after unprotected anal intercourse with an unknown male partner in Germany. He had genital herpes in his personal history and no other sexually transmitted infections. Physical examination revealed multiple well-circumscribed deep-seated firm papules with central umbilication on the pubic area and the shaft of the penis. Lesions were relatively the same size and same development stage, surrounded by an erythematous halo, followed by swollen lymph nodes in the groin. Physical examination revealed no other skin/mucous lesions. The patient was otherwise healthy. Serological tests for syphilis (Venereal Disease Research Laboratory – VDRL and Treponema Pallidum Hemagglutination Assay – TPHA) and HIV were negative. He was treated with a single oral dose of azithromycin 1 gram and the next day the fever disappeared. By the fourth day, lesions had a central crust (Fig. 1) and were still painful. Since sexually transmitted diseases had been excluded and monkeypox was suspected the patient was referred to the Clinic for Infectious Diseases where skin lesions swabs were collected, and a real-time polymerase chain reaction detected monkeypox virus. He was treated with symptomatic therapy and hospitalized for five days. After that, he was isolated at home. All skin lesions regressed completely within 5 weeks.

**Figure 1:**
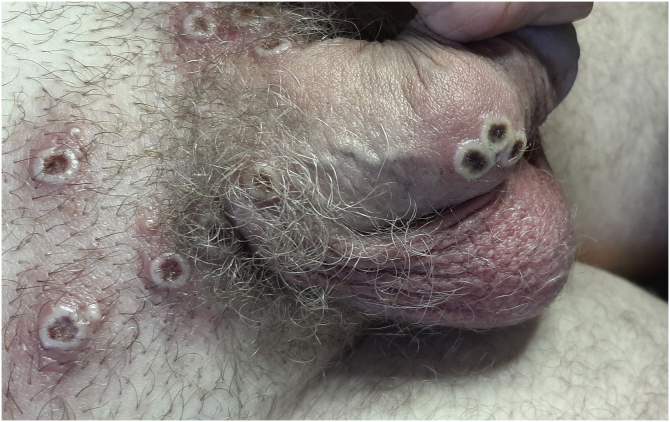
Multiple crusted lesions in the genital region.

The incubation period of the Monkeypox virus infection is one to two weeks. Shortly after the prodrome (fever, headache, malaise, lymphadenopathy) a rash with centrifugal distribution (face, arms, legs, including palms and soles) appears going through different stages (papules, vesicles, pustules, scabs) before healing. Infection can be transmitted through respiratory droplets, close physical contact, including sexual intercourse, and through contact with fomites.^4^, ^6^ Patients are infectious once symptoms begin and remain infectious until all skin lesions have resolved.

The outbreak of monkeypox in the European region is characteristic of males between 31 and 40 years old, predominantly among men who have sex with men.^5^ Although cases outside endemic regions are often linked to international travel, in this outbreak there are no travel links to Africa. Human-to-human transmission occurs by direct contact including during sex, as we described in our case. The clinical presentations in this outbreak are unusual with lesions in the ano-genital region and mild prodromal symptoms. Hence, the disease could be confused with sexually transmitted infections (secondary syphilis, genital herpes, chancroid) or varicella-zoster virus infection.^4^ This stresses the importance that dermatologists at outpatient clinics identify and isolate cases early and promptly trace contacts. Dermatologists should remain vigilant, although monkeypox is not a classic venereal disease, it could be transmitted during sexual intercourse.

## Financial support

None declared.

## Authors' contributions

Milan Bjekic: Approval of the final version of the manuscript; intellectual participation in propaedeutic and/or therapeutic management of studied cases; preparation and writing of the manuscript; study conception and planning.

Milica Markovic: Critical literature review; data collection, analysis, and interpretation; intellectual participation in propaedeutic and/or therapeutic management of studied cases; manuscript critical review.

Lidija Dejanovic: Effective participation in research orientation; data collection, analysis, and interpretation; manuscript critical review.

## Conflicts of interest

None declared.
